# Reliability of an adapted core strength endurance test battery in individuals with axial spondylarthritis

**DOI:** 10.1007/s10067-020-05408-6

**Published:** 2020-09-21

**Authors:** Anne-Kathrin Rausch, Philipp Baltisberger, André Meichtry, Beatrice Topalidis, Adrian Ciurea, Theodora P. M. Vliet Vlieland, Karin Niedermann

**Affiliations:** 1 School of Health Professions, Institute of Physiotherapy, Zurich University of Applied Sciences, 8401 Winterthur, Switzerland; 2 Department of Orthopaedics, Rehabilitation and Physical Therapy, Leiden University Medical Center, Leiden, Netherlands; 3 Ankylosing Spondylitis Association of Switzerland, 8050 Zurich, Switzerland; 4 Department of Rheumatology, Zurich University Hospital, 8091 Zurich, Switzerland

**Keywords:** Ankylosing spondylitis, Assessment, Group exercise, Physical therapy

## Abstract

**Objectives:**

To adapt the core strength endurance test battery (aCSE), previously used for testing athletes, to a target group of patients with axial spondylarthritis (axSpA), to evaluate its intra-tester reliability and its associations with disease-specific factors.

**Methods:**

A cross-sectional study was conducted at axSpA exercise therapy groups, including both axSpA patients and the physiotherapist group leaders (PTs). The aCSE was used to measure the isometric strength endurance of the ventral, lateral, and dorsal core muscle chains (measured in seconds), as well as to assess the disease-specific factors of functional status, self-reported pain, and perceived strength performance. The aCSE was repeated after 7–14 days to measure intra-tester reliability for the same rater (PT group leader). Reliability was calculated as an intra-class correlation coefficient (ICC) using a nested design. The associations between ventral, lateral, and dorsal strength endurance and the disease-specific factors were calculated using Pearson correlation coefficients.

**Results:**

Study participants were 13 PT group leaders and 62 axSpA patients. The latter were all capable of performing the aCSE, with the exception of one individual. A moderate to substantial intra-rater reliability (ICCs (95%CI)) was found for the ventral (0.54 (0.35, 0.74)), lateral (0.52 (0.33, 0.70)), and dorsal (0.71 (0.58, 0.86)) core muscle chains. None of the aCSE measures correlated with the disease-specific factors.

**Conclusion:**

The aCSE was found to be a reliable test battery for assessing core strength endurance in axSpA patients. Interestingly, aCSE performance was not associated with any disease-specific factors.

**Table:** 

**Key Points** • *The adapted core strength endurance test battery measures the isometric strength of the ventral, lateral and dorsal core muscle chains.* • *The adapted core strength endurance test battery showed a moderate to substantial intra-rater reliability for all three muscle chains tested in axSpA patients.* • *No correlations were found between the adapted core strength endurance test battery and the disease-specific factors of self-reported pain, functional status and perceived strength performance.*

**Electronic supplementary material:**

The online version of this article (10.1007/s10067-020-05408-6) contains supplementary material, which is available to authorized users.

## Introduction

Axial spondylarthritis (axSpA) is a chronic inflammatory rheumatic condition that mainly affects the axial skeleton, iliosacral joints, and spine. It can lead to structural and functional impairments and can have a significant impact on an individual’s quality of life and working ability [1, 2].

The cornerstones of non-pharmacological treatment are patient education and regular exercise [1]. Current recommendations for physical activity in individuals with inflammatory arthritis emphasize that the general physical activity recommendations, comprising the four exercise domains of cardiorespiratory fitness, muscle strength, flexibility, and neuromotor performance, are effective, safe, and feasible for axSpA patients [3].

In addition to peripheral muscle strength, core muscle strength (sometimes referred to as the “powerhouse”) is especially important for people with axSpA [4], since inflammation and reduction in mobility affect the dynamic stabilization of the spine. A certain level of core stability is needed, in terms of strength and muscle fatigue resistance, for the activities of daily living (improved posture, enhanced balance, and proprioception) and sports performance (such as correct and safe barbell-based exercising) [5]. Core muscle performance is a complex and multivariable construct, with core stability not yet having been clearly defined [6, 7]. Experts agreed in a Delphi-project that core stability is “the ability to achieve and sustain control of the trunk region at rest and during precise movement” and depends on the components of muscle strength and neuromotor control [8]. Three common categories to assess core muscle strength can be differentiated according to this concept: maximal strength, power [9, 10], and strength endurance, the latter defined as the ability to sustain a given level of force production over time [11].

No randomized controlled studies have been published to date investigating the effect of muscle strength exercises alone on lower limb and trunk muscle strength performance in axSpA patients [12]. Training the trunk muscles does seem, however, to have a positive effect on flexibility in healthy people [13]. Moreover, the combination of core strength and flexibility exercises has been found to have a positive effect on disease activity and flexibility in axSpA patients (assessed by the Bath Ankylosing Spondylitis Mobility Index, BASMI) [14].

The physical activity recommendations for individuals with inflammatory arthritis [3] underline the fact that regular evaluation of physical fitness, including all afore mentioned four domains, should be part of standard care for axSpA patients [3]. The assessment of strength, however, is challenging. Dagfinrud et al. found that the monitoring of muscle strength parameters in group exercise therapy (GET) studies was essentially nonexistent [15]. A survey of the axSpA GET in Switzerland and the Netherlands confirmed this finding for clinical practice [16]. However, there is a need to regularly assess all fitness dimensions to identify those with the potential for improvement. This could help healthcare providers and patients in the process of promoting physical activity, e.g., giving advice, setting goals, planning, and performance of exercises.

The Ankylosing Spondylitis Association of Switzerland (Schweizerische Vereinigung Morbus Bechterew, SVMB) currently has more than 4000 members and organizes more than 60 exercise groups across Switzerland. Participants exercise weekly in groups led by physiotherapists (PTs) in land-based or water-based settings. The objectives of the GET are to minimize the progressive spinal mobility restriction [17], reduce cardiovascular and biological risk factors [18], and maintain or increase muscle strength. According to the recommendations [3], annual fitness assessments are also part of the SVMB’s quality concept. In clinical settings, such as the axSpA GET, the fitness dimensions should be assessed by easy-to-use and inexpensive devices. In this target group, strength assessment should focus on core muscle strength endurance rather than peripheral muscle strength.

In the absence of an existing gold standard, the isometric core strength endurance test battery (CSE), originally developed by the Swiss Olympic Medical Centers for use with athletes, seems to be the only available tool for use in an axSpA GET setting. It was found to be easy to perform, inexpensive to use, and to present with good psychometric properties [19]. The Biering-Sorensen test, which is part of the aCSE, showed good to excellent inter-rater and test-retest reliability [20], but no prior data on intra-rater reliability were found. The CSE was designed to evaluate the “basic” core strength of athletes, meaning the minimum strength required for the performance of sports [19]. With the aid of a reference table including norm data of Swiss athletes, assessors judge whether core strength is sufficient or insufficient.

The aim of this study was to adapt the CSE for axSpA patients (aCSE), evaluate intra-tester reliability and the associations with disease-specific factors.

## Methods

### Design

For the analysis of reliability, a cross-sectional study with a nested design was conducted, given that across the groups, a different numbers of individuals were measured by the PTs (raters). The findings are reported in line with the GRRAS (Guidelines for Reporting Reliability and Agreement Studies) guidelines [21].

### Participants

#### Physiotherapists (PT)

The PTs leading the GET in the German-speaking region of Switzerland (*n* = 45) were invited to participate in this study and to perform and rate the aCSE test battery with their group participants. They were requested to attend a 2-h practical training session (lead by PB). In addition, they received detailed step-by-step explanations in the form of videos, photos, and handouts. Thirteen PTs registered for the aCSE training.

#### AxSpA patients

An information letter was sent to the 206 participants of the SVMB exercise groups of the 13 PTs registered for the aCSE training. The letter explained the purpose and procedures of the study and described the aCSE test battery. Inclusion criteria were age of over 18 years and sufficient German language skills. Eligible individuals also had to be capable of getting down onto the floor and lying in the prone, supine, and side positions. Exclusion criteria were heart diseases of class three or four, according to the New York Heart Association [22], diagnosed osteoporosis of grade two or more [23], or surgical spondylodesis performed on the entire lumbar spine.

### Assessments

The aCSE test battery was performed by each axSpA patient twice with a period of 1 to 2 weeks between the tests (T0 and T1). A period of 1 week allowed for full recovery of the muscles after performing the first aCSE test battery at T0, while the maximum of 2 weeks minimized the possible effect of disease-related changes. The measurements took place during the regular GET. At T0, as well as recording patient characteristics, measurements of pain status, disease activity functional restriction, and physical fitness were made. The same group PT leader conducted the aCSE measurements at both time points with each patient. No encouragement (in terms of cheering) was allowed.

### Participant characteristics

Gender, age, disease duration, and self-reported exercise hours per week were recorded for axSpA patients. Gender, age, and work experience in years were documented for the PTs.

### Core strength measurements

#### Adaptation and performance of the core strength endurance test battery (aCSE)

Given the large number of exercise groups that could potentially implement the aCSE, the criteria for test selection were validity, reliability, and low cost. A further important criterion was feasibility, i.e., the test had to be able to be performed in sports halls with little specific equipment and by individuals with a broad range of fitness and health.

Previous field testing had shown that the CSE used with athletes [19] was too demanding for some SVMB exercise group participants. They reported joint pain while in positions of lateral and ventral muscle chains. The following adaptations were consequently made to the CSE for testing axSpA patients, resulting in an adapted version named the aCSE:

- The starting position for the ventral plane was changed from plank to quadruped position.
- Tests were performed statically instead of dynamically [24] to ensure a constant load (this modus reduced the risk of injury and was easier to standardize).
- Rods were used for standardization, i.e., the participant was asked to keep contact with the horizontal rod during the test (see supplement, Fig. 1).

The time that the subject was able to remain in each of three positions was measured in seconds. Time recording stopped whenever the participant lost contact with the rod for the third time. The participant could get into the required position for a maximum of three attempts. Before the next position was measured, the participant rested for at least 30 s. Detailed instructions on the performance of aCSE can be found in the Supplement.

### Additional outcomes and assessments

#### Pain intensity

Pain intensity was measured using the Numeric Rating Scale (NRS), which is acknowledged as a reliable and valid measurement tool in clinical practice and research [25]. The NRS measures pain on a scale from 0 to 10 (0 = no pain at all and 10 = worst pain ever). In this study, the tool was used to assess average pain immediately before and after the aCSE test batteries were performed.

#### The Bath Ankylosing Disease Activity Index (BASDAI)

AxSpA disease activity was measured using the BASDAI, which is a valid and reliable self-reporting questionnaire [26]. The questionnaire consists of six items to determine pain in the peripheral joints and spine, fatigue, morning stiffness, and pain sensitivity to touch [27]. The BADAI results in a mean score of 0–6 points (0 = no disease activity, 6 = highest disease activity) [27].

#### The Bath Ankylosing Spondylitis Functional Index (BASFI)

Functional limitations experienced by axSpA patients during ten everyday tasks were evaluated using the BASFI, which is a valid and reliable self-reporting questionnaire [26]. The BASFI evaluation results in a mean score between 0 and 10 (0 = no handicap and 10 = highest possible degree of functional limitation).

#### Physical fitness questionnaire (FFB-Mot.)

Self-perceived motor performance ability during everyday tasks, with regard to cardiorespiratory fitness, strength, flexibility, and coordination, was evaluated using The Physical Fitness Questionnaire (FFB-Mot., German: Fragebogen zur Erfassung des motorischen Funktionsstatus) [28]. It has been shown that the FFB-Mot. is a valid instrument to determine physical fitness in a healthy adult population [29]. The outcome of the 28 items on the questionnaire results in a total score ranging from 5 to 140; the strength questions include seven items for which the score can range from 0 to 30. For example, a total score of 35/140 for a 58-year-old healthy man would indicate a rather poor overall physical fitness status, while a score of 100/140 would indicate a rather good physical fitness status [29]. It has not yet been used as an assessment for evaluation, so no reliability data are available.

### Weekly exercise

Questions on weekly hours of planned exercise, together with the focus of the exercise dimension (muscle strength, cardiovascular, neuromotor, or flexibility), were asked by the PT, together with the patient characteristics (age, gender, year of diagnosis, and disease duration).

### Sample size and statistical analysis

Sample size was estimated based on the precision of the reliability estimate following Giraudeau and Mary [30]. With a targeted width of the confidence interval (two margin of errors or four standard errors) of *w* = 0*.*3, 57 subjects were needed for (a conservative) ICC = 0.65. For larger ICCs, the required sample size would be smaller or the precision in the analysis would be larger.

All statistical analyses were performed using the R statistical software R version 3.5.3 [31].

#### Reliability

Generalizability theory [32] was used as the framework to estimate the reliability of the raters’ time-keeping while assessing core muscle strength. To estimate the intra-rater reliability, a linear mixed model for “(strength endurance) Time” *Y* of Subject *i* nested in Rater *j* measured at Time point *k* was fitted to the data:

$$ {Y}_{i(j),k}=\mu +S{(R)}_i+{R}_j+{T}_k+{RT}_{ik}+{\epsilon}_{i,j,k} $$

with *Y*_*i*(*j*), *k*_representing strength endurance time, *μ* representing the global mean, S(R)_i_ corresponding to S_i_ + S(R)_ij_ which cannot be disentangled in a nested design, and ε_ijk_ the independent and normal distributed errors.

The intra-rater reliability was calculated as the intra-class correlation coefficient

$$ corr\ \left({Y}_{i(j),k},{Y}_{i(j),{k}^{\prime }}\right)=\frac{\sigma_{S\ (R)}^2}{\sigma_{S(R)}^2+{\sigma}_T^2+{\sigma}_{RT}^2+{\sigma}_{\epsilon}^2}, $$

with the *σ*^2^ representing the corresponding variance components. Bootstrapped confidence intervals for the intra-class correlation coefficient (ICC) were computed. Values less than 0.2 demonstrate a slight reliability, values between 0.2 and 0.39 indicate a fair reliability, values between 0.4 and 0.59 describe moderate reliability, values between 0.6 and 0.79 indicate substantial reliability, and values greater than 0.80 indicate almost perfect reliability between measurements [33]. The lower limit of the 95% confidence interval of the ICC to estimate the level of reliability was used [34].

#### Associations between the aCSE outcomes and disease-specific factors

To evaluate the relationship between aCSE outcomes and patient-reported strength and the disease-specific outcomes, the time measurements for aCSE ventral, lateral, and dorsal were correlated with the FFB-Mot. subscales of strength, NRS-measured pain, BASDAI, and BASFI, using the Pearson correlation coefficient. The size of a correlation coefficient can be interpreted as negligible (*ρ* < 0.3), low (0.3 < *ρ* < 0.5), moderate (0.5 < *ρ* < 0.7), high (0.7 < *ρ* < 0.9), and very high (*ρ* > 0.9), with both positive/negative correlations [35]. A priori, it was hypothesized that the following would provide evidence of an association between aCSE performance and disease-related factors: (a) a positive Pearson correlation (r_s_) > 0.3 between aCSE and FFB-Mot. subscale strength and/or self-reported hours exercise per week; and/or (b) a negative correlation < 0.5 between aCSE and pain (NRS) and/or disease activity (BASFI, BASDAI). The lower limits of 95% confidence intervals adjusted for multiple testing were used.

## Results

Thirteen PTs (28.8%) and 62 group participants (30.0%) provided informed consent and were included in the study; three of the latter could not participate at T1 (*n* = 2 due to acute influenza, *n* = 1 due to pain after the first measurement). Descriptive data for the study participants are shown in Table 1.

**Table 1:** Participants’ characteristics

Characteristics of axSpA patients	*n* = 62
Gender, female, *n* (%)	29 (46)
Age, years	54.6 [11.2]
BASDAI (0–10)	3.5 [2]
BASFI (0–10)	1.8 [1.5]
FFB-Mot. total (0–140)	85.8 [18]
FFB subscale strength (0–35)	21.5 [5.4]
NRS pre aCSE at T0	2.2 [2.2]
NRS post aCSE at T0	1.6 [2.4]
Disease symptoms since, years	27.5 [12.9]
Disease duration since diagnosis, years	20.1 [13]
Self-reported training per week, hours	3.1 [2.9]
Characteristics of physiotherapists	*n* = 13
Gender, female, *n* (%)	9 (69)
Age, years	46 [11]
Work experience, years	6.3 [3.5]
Number of participants per rater (median, range)	4 [2–9]

Data are mean (standard deviation), if not stated otherwise. *BASDAI*, Bath Ankylosing Disease Activity Index; *BASFI*, Bath Ankylosing Spondylitis Functional Index; *FFB-Mot*., physical fitness questionnaire; *NRS*, numeric rating scale; *aCSE*, adapted core strength endurance test battery; *T0* and *T1*, time point 1 (baseline) and 2, respectively; *GET*, group exercise therapy

### Intra-rater reliability of aCSE

A moderate to substantial intra-rater reliability was found for all three test positions that tested the strength of the ventral, lateral, and dorsal core muscle chains (Table 2).

**Table 2:** Intra-rater reliability of the aCSEs

Test position	ICC	95% CI
aCSE ventral	0.54	0.35, 0.74
aCSE lateral	0.52	0.33, 0.70
aCSE dorsal	0.71	0.58, 0.86

*aCSE*, adapted core strength endurance test battery; *CI*, confidence interval; *ICC* intra-class correlation coefficient

### Associations of aCSE outcomes with disease-specific factors

Data shown in Table 3 indicate no positive or negative associations between aCSE outcomes and any disease-specific factors measured. Thus, the a priori hypotheses were not confirmed.

**Table 3:** Evaluation of associations between aCSE and disease-specific factors

Association	*r*	95%CI*
aCSEv vs. FFB-Mot. strength	0.25	− 0.19, 0.61
aCSEl vs. FFB-Mot. strength	0.34	− 0.11, 0.67
aCSEd vs. FFB-Mot. strength	0.30	− 0.15, 0.65
aCSEv vs. self-reported training hrs	− 0.06	− 0.44, 0.34
aCSEl vs. self-reported training hrs	− 0.06	− 0.44, 0.34
aCSEd vs. self-reported training hrs	− 0.01	− 0.32, 0.30
aCSEv vs. pain	− 0.11	− 0.50, 0.31
aCSEl vs. pain	− 0.07	− 0.44, 0.33
aCSEd vs. pain	0.06	− 0.33, 0.43
aCSEv vs. BASFI	− 0.03	− 0.39, 0.33
aCSEl vs. BASFI	− 0.16	− 0.54, 0.27
aCSEd vs. BASFI	− 0.35	− 0.68, 0.10
aCSEv vs. BASDAI	− 0.23	− 0.59, 0.22
aCSEl vs. BASDAI	− 0.26	− 0.62, 0.19
aCSEd vs. BASDAI	0.03	− 0.33, 0.37

*r*, observed correlation coefficient; *CI*, confidence interval (*uncertainty adjusted for multiple testing (Holm procedure)); *BASDAI*, Bath Ankylosing Disease Activity Index; *BASFI*, Bath Ankylosing Spondylitis Functional Index; *FFB-Mot*. strength, physical fitness questionnaire subscale strength; *NRS*, numeric rating scale; *aCSE*, adapted core strength endurance test battery; *v*, ventral; *l*, lateral; *d*, dorsal

## Discussion

The aCSE values for the intra-rater reliability of the ventral, lateral, and dorsal planes show a moderate to substantial level of agreement. The results show that PTs are able to use the aCSE reliably, even with little experience of aCSE testing. Furthermore, nearly all participants were able to perform the aCSE, and only one (1.6%) was unable to. In conclusion, the aCSE is a reliable assessment for people with axSpA in a group setting.

Possible associations between aCSE performance and disease-specific factors, according to the a priori hypotheses, were not confirmed. This is an interesting finding, suggesting that the aCSE can be performed by an individual with axSpA irrespective of their perceived strength performance, functional status, and self-reported pain. This may be an aspect worthy of further investigation.

However, we were unable to confirm that pain did not influence aCSE performance. Pain was reported using NRS prior to and post-testing, but no statistically significant negative relationship was found that would imply that more pain is related to less strength. Other than pain [36], the factors of motivation and effort are determinants of strength performance [37]. Midgley and colleagues hypothesized that verbal encouragement could make a difference to test outcomes, through having a positive impact on intrinsic motivation and physical performance [38]. The rating of perceived exertion together with consistent verbal encouragement should be incorporated into the future use of the aCSE. Consistent verbal encouragement could be usefully included in the GET assessment situation.

Due to the complexity of the core strength construct, there has been no gold standard for assessing core strength in the past, or core strength endurance in particular. Core strength is vital for maintaining an upright posture and is especially important for axSpA patients, who are affected by spinal inflammation and decreased spinal mobility. It is therefore appropriate to assess core strength endurance for axSpA patients. The physical position for assessing these individuals poses an additional challenge. It could be argued that to assess strength endurance of the dorsal muscle chain, the testing position described by Ito and colleague [39] (lying in prone position performing back extension) would be preferable to the testing position described by Biering Sorensen [40] (lying in prone position half of the body fixed on a treatment bench holding neutral position), because the Biering Sorensen occasionally activates more hip extensor muscles [24, 41]. However, since the restricted flexibility of the spine is a major impediment in axSpA patients, the test procedure described by Biering Sorensen was considered to be more appropriate for covering the complete range of impairments from low to high. In future, it would be useful to develop a score for strength that encompasses all three planes, or to investigate whether there is a score from one plane that would serve as a sufficient proxy for all three planes. Ultimately, a database containing norm core strength values of individuals both with and without axSpA could ensure a meaningful interpretation of test results.

The original CSE is used as part of a sports performance test battery for athletes [19]. With the aid of a reference table containing norm data of Swiss athletes, assessors judge whether core strength is sufficient or insufficient. Currently, no reference table including norm data of people with axSpA is available. This aspect should be taken into consideration when reporting test results to participants. However, until such a set of norm data is established, intra-individual comparisons might give an orientation.

Surprisingly, no relationship between aCSE outcomes and perceived strength performance was found, although there is evidence that people of all ages have a moderately accurate perception of their physical fitness [42]. In contrast, other studies [43] provide evidence that people tend to be unrealistic in assessing their abilities, such as physical fitness or level of physical activity. A further reason for our finding could be that the aCSE measures core stability and not strength endurance. Core stability was not explicitly assessed in the patient-reported outcome measure. However, there is a consensus that core stability is dependent on the components of muscle strength and neuromotor control [8]. Majewski-Schrage and colleagues [8] asked experts to provide assessment techniques that were specific to the components of core stability. The top three answers were timed side bridge, Sorenson test, and timed prone bridge. All three of these are elements of the aCSE.

Low-cost was prioritized in our study setting. However, future projects could investigate the use of appropriate, objective, strength assessment devices, such as a hand-held dynamometer [44]. This has been used previously to measure core strength indirectly (albeit maximal strength, not strength endurance) and has been found to be reliable and valid [7, 45]. It might be less time-consuming to incorporate, e.g., easy to perform handgrip, instead of core strength, especially in a group setting.

This study has strengths and limitations. To our knowledge, this was the first study to evaluate the psychometric properties of a core strength assessment in axSpA patients. Movement assessment by visual observation and time-keeping appears to be feasible for PTs. However, it is a limitation of this study that reliability was tested by the clinically working usual GET PT leaders rather than by raters in a laboratory setting. Although the 2-h assessment training was designed to standardize the test procedures and was appreciated by the PTs, their performance was likely to have been influenced by their motivation and understanding of the importance of performing the aCSE in a standardized way. PTs were not asked to perform the additional, disease-specific assessments, such as the Bath Ankylosing Mobility Index (BASMI), since the participants needed to receive specific instructions on how to hold the correct positions and to exert themselves. Additionally, the PTs emphasized that it was challenging to perform the assessments with each individual in the group during the usual GET sessions, in terms of time management and the planned exercise program. These factors (i.e., PTs’ adherence to standardization, PTs’ motivation, complexity of time-consuming tests) may have had an impact on the accuracy and reliability of the measurement outcomes. The reliability values could potentially be improved with improved test conditions.

## Conclusion

Regular fitness assessment on an individual basis is part of the SVMB’s concept of quality and is recommended [3]. The choice of assessment used in this study was influenced mainly by feasibility criteria, such as low-cost, easy-to-use for axSpA patients, and the ability for people with different health and fitness status to perform in both land-based and water-based GET settings. Appropriate assessments with good psychometric quality are necessary. In summary, the aCSE was found to be a feasible assessment instrument to measure core strength in axSpA patients reliably. It could be potentially combined with other assessments of aerobic fitness, flexibility, and neuromotor performance to establish an inexpensive and practical assessment battery, covering all exercise dimensions, for axSpA patients. Future research should establish a reference norm data table including axSpA patients, to enable an appropriate interpretation of test results. In addition, a less time-consuming alternative to implementing aCSE testing should be investigated, e.g., using only one representative plane instead of three separate planes.

## Electronic supplementary material

ESM 1 (DOCX 81 kb)
