# Supplementary material for: Reliability of an adapted core strength endurance test battery in individuals with axial spondylarthritis
Source: Clin Rheumatol. 2020 Sep 21;40(4):1353–60. doi: 10.1007/s10067-020-05408-6 (PMC7943491; doi:10.1007/s10067-020-05408-6)
Supplement: Supplementary file 1 — (DOCX 81 kb) [file 10067_2020_5408_MOESM1_ESM.docx]

*Performance of aCSE ventral plane*

The participant was asked to take up a quadruped position where the feet rest on a 15cm diameter Pilates roll, the knees were located underneath the hips, and the hands were positioned underneath the shoulder joints. Then, the participant was instructed to lift his/her knees from the mat (to a maximum of 10 cm), where the rater placed the rod at the subject’s sacrum (Figure 1). The participant was asked to remain in this position as long as possible and stay in contact with the horizontal rod.

*
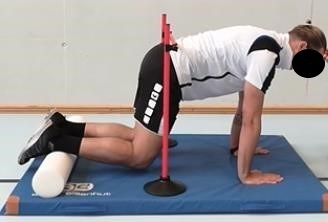
* Figure 1: aCSE ventral plane

*Performance of aCSE lateral plane*

The participant performed an adapted side plank position on the preferred side (only one side was measured), with the arms crossed in front of the body and the feet resting on a 15cm diameter Pilates roll or box, while the horizontal rod was placed at the greater trochanter of the hip (Figure 2). The participant was asked to remain in this position as long as possible and stay in contact with the rod.

*
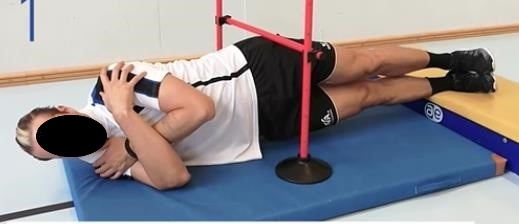
* Figure 2: aCSE lateral

*Performance of aCSE dorsal*

The dorsal side was measured while the participant was lying on a gymnastic box (e.g. Alder-Eisenhut like on Figure 3) positioning the spinae iliaca anterior superior congruent with the edge of the box. The legs of the participant were fixed on the gymnastic box with two manual traction belts. The participant was asked to take up his/her best possible active achievable position for extension movement of the spine and to remain in the given position as long as possible while the horizontal rod was placed at the scapulae (Figure 3).


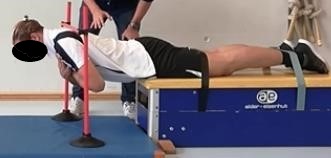
Figure 3: aCSE dorsal
